# Left atrial longitudinal strain analysis in long Covid-19 syndrome

**DOI:** 10.1007/s10554-023-02801-5

**Published:** 2023-02-14

**Authors:** Shimaa Gamal ZeinElabdeen, Abdelsalam Sherif, Nader Talaat Kandil, Abdalaali Mohammed Omar Altabib, Mahmoud Abdelaziz abdelrashid

**Affiliations:** 1 Cardiology Department, Faculty of Medicine, Zagazig University, Zagazig, Egypt; 2 Faculty of Medicine, Alzawia University, Zawia, Libya

**Keywords:** COVID-19, Left atrial strain, Left ventricular diastolic dysfunction, LA stiffness Long COVID-19 syndrome

## Abstract

It is known that during the active course of Coronavirus disease 2019 (COVID-19), myocardial injury has an established pathological base, while its myocardial injury post-recovery is still obscured.The aim of this study was to evaluate the longitudinal left atrial strain (LAS) using speckle tracking echocardiography (STE) in COVID-19-recovered patients who are previously healthy without confounder comorbidities to detect the potential cardiac dysfunction.200 patients were prospectively included and examined 4?12 weeks after recovery from COVID-19 infection. 137 participants with comorbidities or previous history of cardiopulmonary disease were excluded from the analysis. A total of 63 patients who fulfilled our inclusion criteria were recruited into two groups according to thepresence or absence of persistent dyspnoea and exercise intolerance. Clinical, laboratory & comprehensive echocardiographic examinations were done for all. We observed that 31.7% of the previously healthy individuals developed dyspnoea & exercise intolerance post-COVID-19 infection. There were significantly impaired LAS parameters in the symptomatic group (LA reservoir, contraction & conduit strain, 22.7%, -6.6% & -16.1% versus 40%, -12%, and ? 27% in the asymptomatic group with P < 0.000). Only LA reservoir strain and LA stiffness can independently predict the development of dyspnoea & exercise intolerance post-COVID-19 at cut-off values of 30% & 24.5% respectively with a sensitivity of 90% and a specificity of 91%, P < 0.001. These impaired LAS parameters could explain the developed symptoms post-COVID-19 recovery, even before disturbed conventional diastolic echocardiographic parameters.LAS parameters are significantly associated with the developed exertional dyspnoea & exercise intolerance post-COVID-19. LA reservoir strain & LA stiffness could provide a simple, easily available tool that points to early LV diastolic dysfunction and may direct the therapy in this subset of the population.

## Introduction

The Coronavirus disease 2019 (COVID-19) pandemic has an established adverse clinical impact on the cardiovascular system. However, little is known about the intermediate & longer-term sequelae of this infection, defined as “long COVID” where persistent COVID-19 symptoms last beyond 12 weeks post-infection [1].

The pathophysiologic basis for the persistent symptoms and functional limitation among patients who have long since recovered from mild acute illness remains unknown. However, Observations point out that this population does not appear to have previous cardiac or pulmonary pathology or any major pulmonary function abnormalities during the acute course of COVID-19 infection [2].

Accordingly, in the current study, we aimed to evaluate subjects who developed exertional dyspnoea, fatigue & exercise intolerance beyond weeks from complete recovery, and who did not show any overt cardiopulmonary residue proved by conventional imaging modalities.

## Methods

### Study population

200 patients were prospectively included and examined 4–12 weeks after recovery from COVID-19 infection. 137 Participants with comorbidities or previous history of cardiopulmonary disease were excluded from the analysis, total of 63 adults > 18 years old patients who fulfilled the inclusion criteria were included in our study and recruited into two groups; group (A) includes 20 symptomatic subjects who developed exertional dyspnoea, fatigue & exercise intolerance (NYHA class ≥ 2), compared to group (B) includes 43 patients without any residual symptoms. All participants were in sinus rhythm, patients with evidence of coronary artery disease, arterial hypertension, diabetes mellitus, left ventricular (LV) wall motion, LV cardiomyopathy, valvular heart disease, dysrhythmia especially atrial fibrillation, atrioventricular conduction abnormalities on ECG, thyroid dysfunction, pulmonary disease, respiratory failure or poor echocardiographic image quality were excluded from the study. Pulmonary embolism secondary to COVID infection was excluded by CT chest scan

### Ethical considerations

This case-control study was approved by our institutional review board (IRB); ZU-IRB#9204/4-1-2022 and conducted at our University Hospital’s cardiology department between October 2021 and October 2022. All procedures were performed following the ethical standards of the national research committee. All participants gave written informed consent to participate in the study.

### Transthoracic echocardiography

The echocardiographic examination was performed using a Vivid E95 (General Electric Health Care, WI, USA) ultrasound machine, and images were acquired with the patient in left lateral decubitus using a 3.5–5 MHz transducer at a depth of 16 cm. ECG was recorded, and 3 consecutive cardiac cycles of each view were recorded during quiet breathing at 50–80 frames/sec. All patients were subjected to conventional transthoracic echocardiography & Speckle tracking echocardiography (STE). All measurements were taken following the American Society of Echocardiography (ASE) recommendations [3].

Left atrium (LA) dimension, left atrial volume index (LAVI) and left ventricular (LV) end-systolic and end-diastolic diameters were measured. LV ejection fraction was estimated by modified Simpson’s rule. Trans mitral pulsed-wave Doppler velocities were recorded from the apical four-chamber view with the Doppler sample placed between the tips of mitral leaflets. Early (E) and late (A) wave peak velocities, E/A ratio, E wave deceleration time (DT), and isovolumetric relaxation time (IVRT) were measured.

Tissue Doppler imaging was recorded during quiet breathing, at a rate of at least 105 frames/ sec. The myocardial early diastolic (e`), and late diastolic (a`) velocities were obtained at lateral & septal mitral annuli. The E/e` ratios were subsequently calculated. Left ventricular diastolic function was assessed and graded according to the recommendations for the Evaluation of Left Ventricular Diastolic Function by Echocardiography [4].

### 2D speckle tracking

Left atrial longitudinal strain analysis was obtained using automated speckle tracking software. The regions of interest (ROI) were generated automatically and LA endocardial border was manually adjusted when required.

LA phases definition and LAS values were measured from the LA longitudinal strain curve according to the European Association of Cardiovascular Imaging (EACVI)/American society of echocardiography (ASE) guidelines [5].

**Fig. 1:**
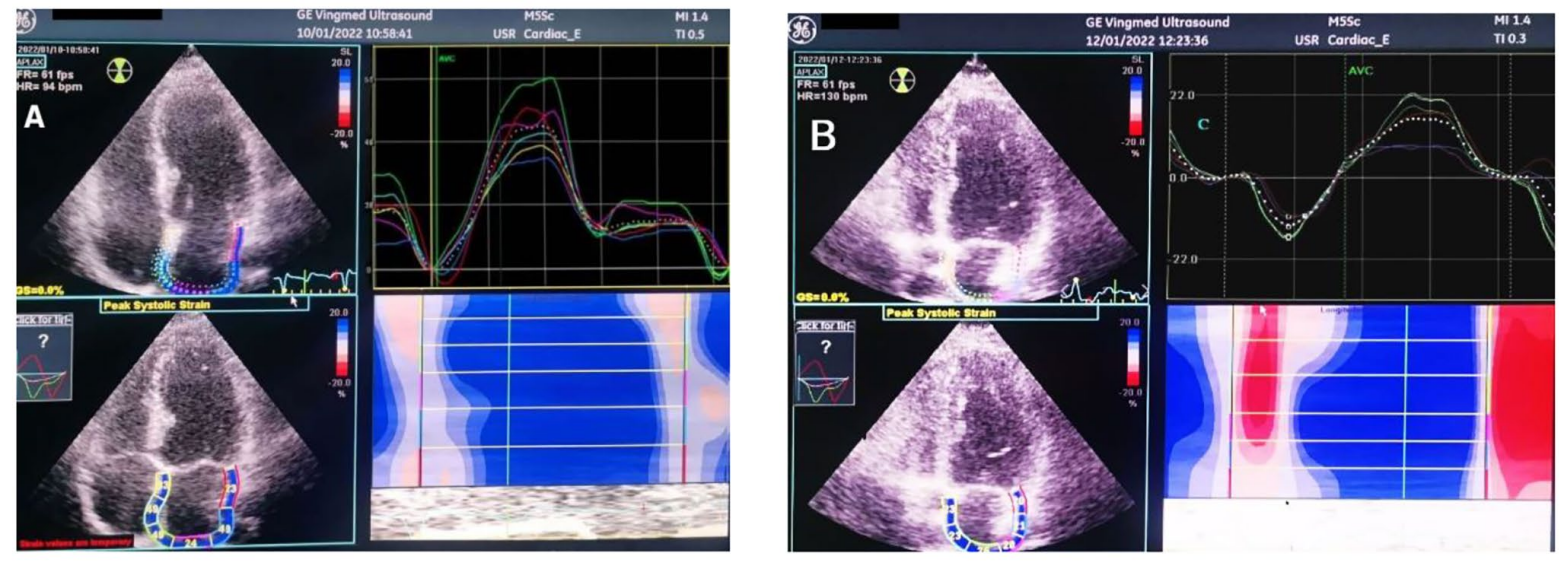
LA speckle tracking strain. **(A) ***showing normal peak longitudinal/ reservoir LAS in an asymptomatic patient, average LAS is 43% ****(B) ****showing reduced peak longitudinal/reservoir LAS in a COVID-19 patient who developed exertional dyspnoea & exercise intolerance. average LAS is 20%.*

LAS analysis was calculated with the reference point set at the onset of the QRS complex of the superimposed ECG, two longitudinal deformation parameters are identified, positive peak atrial longitudinal strain at the end of the reservoir phase and a negative peak atrial contraction strain, before atrial contraction [6]. (Fig. 1.

**Fig. 2:**
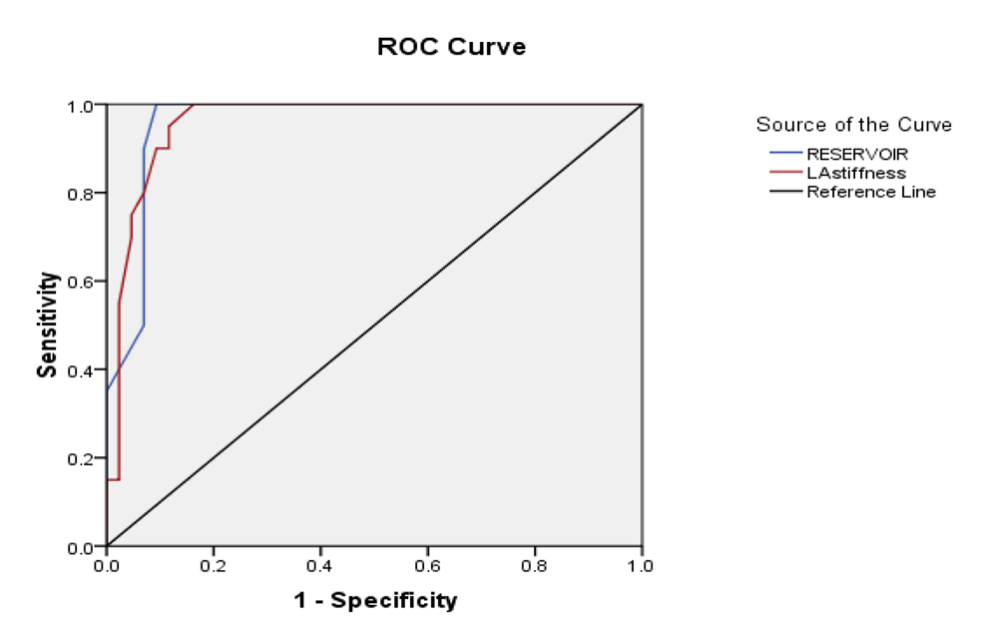
ROC curve: Predictors of LV diastolic dysfunction post COVID-19

LA strain rate was measured during the early ventricular filling phase. LA stiffness was calculated as the ratio of E/eʹ to LA reservoir strain x100 [7].

Global LV systolic strain (GLS) was evaluated, and the software automatically traced the contour of the endocardium at apical three, four and two-chamber views. 2D GLS was analysed during aortic valve closure.

### Statistical analysis

Data are expressed as mean standard deviation (SD) for continuous variables, and categorical variables, as numbers & percentages. The student-t test and Chi-square test were used to compare variables between groups. To evaluate the diagnostic performance of left atrial strain and LV diastolic function parameters for the prediction of cardiac affection, a receiver-operating characteristic curve (ROC) was constructed. Multivariate Logistic regression analysis was adjusted for age, sex, and BMI to evaluate predictors of persistent dyspnoea post-COVID-19 infection. Data are presented as odds ratios (ORs) and 95% confidence intervals (CIs). A statistical test was significant when the P value was under 0.05. All P values are the results of 2-tailed tests. Statistical analyses were performed using SPSS software version 16.

## Results

Between October, 2021, and October, 2022, 200 patients who recovered from the COVID-19 infection course were followed up routinely at our outpatient clinic for clinical and transthoracic echocardiographic evaluation. 137 patients did not match our inclusion criteria & were excluded while a total of 63 participants were included in our study. According to the presence of exertional dyspnoea & exercise intolerance, two groups (symptomatic and asymptomatic) were recruited.

We observed that dyspnoea & exercise intolerance prevalence was 31.7% post-COVID-19 in our cohort, while sinus tachycardia was present in 33% of them. No differences were observed in age, sex, BMI, BSA or basal CRP level in the acute stage between groups (Table 1).

**Table 1:** Demographic & clinical characteristics of studied groups

VARIABLE	Group A N = 20	Group B N = 43	P value
Age (years)	26 ± 3.7	27.6 5.9	0.782
Sex			
MALE	11 (55%)	19 (44.2%)	0.589
FEMALE	9 (45%)	24 (55.8%)	
BMI (kg.m^2^)	25.9 ± 4.5	24 ± 4.7	0.075
BSA m^2^	1.8 ± 0.3	1.66 ± 0.35	0.064
HR (bpm)	90 ± 13	70 ± 11	0.001^*^
CRP ml	15.6 ± 5.1	12 ± 7.7	0.225
Sinus tachycardia	11 (55%)	10 (23.3%)	0.013
NYHA class ≥ II	20 (100%)	(0%)	0.001^*^

BMI; body mass index, BSA; body surface area, HR; heart rate, CRP; C reactive protein, NYHA; New York Heart Association Classification.

Regarding conventional echocardiographic parameters between groups, we found significantly higher values of E/e` at the septal mitral annulus and IVRT in the symptomatic group, which did not rise to meet the criteria for diastolic impairment, but this shed the light on utilizing an advanced tool for further assessment (Table 2).

**Table 2:** Conventional Echocardiographic parameters of studied groups.

Variable	Group A N = 20	Group B N = 43	P value
ESD mm	23 ±1.9	22.6 ± 1.9	0.45
EDD mm	43 ± 2.2	43 ±1.4	0.52
EF %	63.5 ±4	64 ±3.3	0.374
Peak PAP	25 ± 2.1	24 ± 1.9	0.623
LAVI (mL/m2)	22.6± 6	21 ±3.1	0.323
E wave ( cm)	81± 17	81 ±15	0.947
E/A ratio	1.27 ±0.33	1.23 ± 0.26	0.642
DT (ms)	181 ± 15	189 ±18	0.475
E/e septal	8.4 ±1.6	6 ±1.2	0.001^*^
E/e lateral	6.6 ± 2	5.9 ± 1.9	0.286
IVRT ms	70 ±12	56 ±6	0.001^*^

ESD; end-systolic dimension, EDD; end-diastolic dimension, EF; ejection fraction, Peak PAP; pulmonary artery pressure. LAVI; left atrial volume index, DT; deceleration time, IVTR; Isovolumetric relaxation time.

After measuring strain parameters using the available speckle tracking software, we found significantly impaired LAS parameters in the symptomatic group (LA reservoir, contraction & conduit strain, 22.7%, -6.6% & -16.1% versus 40%, -12% and, -27.5% at the asymptomatic group with P < 0.001). While the left ventricular systolic & diastolic global strain showed no significant difference between both groups as well as was the left atrial diastolic strain (p-value = 0.565, 0.187 & 0.069 respectively) (Table 3).

**Table 3:** Speckle tracking strain echocardiographic data.

Variables	Group A	Group B	P value
LA reservoir strain %	22.7 ± 6	40 ±7.5	0.001^*^
LA contraction strain%	-6.6 ± 2	-12 ±3.7	0.001^*^
LA conduit strain %	-16.1 ± 6	− 27.5 ±7	0.001^*^
LA stiffness%	33.4 ± 8	16.6 ± 6	0.001^*^
LV GLS %	19%	20%	0.565
LA diastolic strain rate s^− 1^	1.9 ± 0.5	2.2 ±1.5	0.187
LV diastolic strain rate s^− 1^	1.7 ± 0.5	1.6 ± 0.3	0.069

LA; left atrium. LV; left ventricle, GLS; global longitudinal systolic strain.

In the Multivariate model analysis (Table 4), after adjustment of age and sex, only LA reservoir strain and LA stiffness can independently predict the development of dyspnoea & exercise intolerance post-COVID-19 at OR of 0.72 (95% CI 0.59 to 0.896) and 1.3 (95% CI 1.034 to 1.68) respectively. These impaired parameters can point to LA myopathy and the subtle Left ventricular diastolic impairment which may explain the developed dyspnoea after recovery from COVID-19.

**Table 4:** Logistic regression analysis of predictors of exertional dyspnoea & exercise intolerance post-COVID-19

	Univariate analysis		Multivariate analysis	
Variables	P value	OR 95.0% C.I.	P value	OR 95.0% C.I.
LAS Reservoir %	0.002^*^	0.68 (0.553–0.843)	0.003^*^	0.72 (0.59–0.896)
LAS Conduit %	0.001^*^	1.32 (1.11–1.55)	NS	
LAS Contraction %	0.001^*^	1.8 (1.33–2.47)	NS	
E/e` septal	0.001*	0.35 (0.190–0.66)	NS	
LA stiffness %	0.001^*^	1.33 (1.15–1.53)	0.001^*^	1.3 (1.034–1.68 )
IVRT ms	0.001*	0.82 (0.72–0.93	NS	
Heart rate	0.001	0.21 (0.160–0.27)	NS	

LA reservoir strain & LA stiffness at cut-off values of 30% & 24.5% respectively with a sensitivity of 90% and a specificity of 91% (95% CI 0.91–1), AUC of 0.959, P < 0.0001 showed the highest diagnostic performance in predicting persistent dyspnoea and exercise intolerance post-COVID-19. (Table 5; Fig. 2).

**Table 5:** Cut-off values and performance accuracy of predictors of persistent symptoms post COVID-19.

Variables	AUC	P value	Cut off value	Sensitivity	Specificity	Asymptotic 95% Confidence Interval	
						Lower Bound	Upper Bound
LA stiffness	0.959	0.001	24.5%	90%	91%	0.913	1.006
LA reservoir strain %	0.959	0.001	30%	90%	91%	0.912	1.006

## Discussion

It is known that the acute viral stressor of COVID-19 may trigger myocardial inflammation either via direct viral invasion in cardiac myocytes or indirect injuries caused by cytokine storm and inflammatory mediators which can potentially affect cardiac structure and function [8].

Vulnerable myocardium in patients with cardiometabolic risk factors can increase the likelihood of myocardial injury during the acute COVID-19 illness or post-recovery, nevertheless, studies evaluating the effect of COVID − 19 on previously healthy, non-vulnerable myocardium are scarce [9].

The background observation for our research was persistent symptoms post-COVID-19 usually start three months from the onset of COVID-19 and last several weeks after complete recovery. These symptoms cannot be explained by obvious pulmonary residual affection and their pathophysiology is not sufficiently evaluated in epidemiological studies [10].

We excluded patients with cardiometabolic risk factors & confounders affecting cardiac function, to assess the direct COVID-19 effect on the myocardium in our population.

Our results showed that 33% of post-COVID-19 patients developed unexplained exertional dyspnoea & exercise intolerance.

This was concordant with other studies that reported that persistent exertional dyspnoea one year after COVID-19 infection was present in more than a third of apparently healthy patients [11, 12]. While higher prevalence was reported by Arnold et al., where persistent breathlessness and excessive fatigue were found in (74%) of their studied patients [13].

Our results showed a statistically significant difference at septal E/e`& IVRT which were higher in the symptomatic group (8.4 & 70 ms) versus (6 & 56 ms) respectively, p < 0.000. Our results come in to agree with previously established studies reported that patients with COVID-19 infection had an increased average E/e′ ratio [14]. However, they found that the majority of patients (80%) did not have elevated LV filling pressure (E/e’≥14).

Also, Szekely et al. revealed a 16% incidence rate of LVDD despite a preserved LV systolic function in 90% of their patients [15].

A previous study used conventional DD (diastolic dysfunction) parameters, performed at 6–10 weeks after hospital discharge, in younger patients, without previous significant cardiovascular disease, they evidenced altered both LV-systolic & diastolic functions in 8.8% of subjects while 16.8% only showed diastolic dysfunction with preserved systolic performance [16].

Many studies are headed to use left ventricular speckle tracking in the detection of subclinical systolic & diastolic dysfunction in COVID-19 [17]. Our results showed normal LV systolic function evaluated by both the conventional volumetric method & global longitudinal strain, this is contrary to studies that showed reduced LV-systolic function in a third of their studied population, which may be due to the severity of COVID-19 infection or confounders affecting the myocardium in their population [18].

Based on the established role of left atrial strain analysis in the detection of LV diastolic dysfunction and its association with abnormal exercise haemodynamic in Heart failure with preserved ejection fraction (HFpEF) [19, 20], we used this tool to evaluate the possible subtle cardiac affection in our cohort. To the best of our knowledge, no African studies were done to ascertain the diastolic dysfunction burden amongst Covid-19 survivors at their intermediate-term utilizing the left atrial strain analysis.

We observed significantly impaired all LAS parameters in patients with persistent dyspnoea post COVID-19 recovery; LA Reservoir, contraction & conduit strain ( -22.7%, 6.6% & 16.1%) versus (-40%, 12% & 27%) with P < 0.000). Similar results showed that hospitalized COVID-19 patients have reduced LA function compared with COVID-19-negative controls and this dysfunction was more pronounced in COVID-19 patients who developed AF [21].

Also, left atrial stiffness (E/e` /LA reservoir strain) showed higher values among the symptomatic group (33.4% versus 16.6% at p < 0.000), which may give a clue for impaired LV lusitropy in this population. At multivariate analysis, we found that only LA reservoir strain and its derived non-invasive LA stiffness parameter were independently associated with the developed symptoms post-COVID-19 at OR of 0.72 (95% CI 0.59 to 0.896) and 1.3 (95% CI 1.034 to 1.68) respectively and are independent predictors for dyspnoea & exercise intolerance occurrence in Long COVID 19 syndrome.

## Conclusion

The observations of our study suggest that LA strain and LA stiffness are early affected in patients with unexplained persistent dyspnoea and exercise intolerance post-COVID-19, and this could be attributed to impaired left ventricular diastolic function. Given the superiority of LA longitudinal strain over conventional diastolic parameters in the prediction of diastolic dysfunction, larger studies with invasive diagnostic modalities are needed to validate our results.

## Limitation

Our results were not compared with an invasive hemodynamic assessment as a gold standard for elevated left ventricular filling pressure, also measurement of NTproBNP could add diagnostic role but, this was practically difficult due to the huge burden of covid-19 recovered patients.

It was a single-centre study with a relatively small sample size secondary to exclusion of numerous clinical confounders that impact the left atrial longitudinal strain.
